# STAT3 Interactors as Potential Therapeutic Targets for Cancer Treatment

**DOI:** 10.3390/ijms19061787

**Published:** 2018-06-16

**Authors:** Federica Laudisi, Fabio Cherubini, Giovanni Monteleone, Carmine Stolfi

**Affiliations:** Department of Systems Medicine, University of Tor Vergata, Via Montpellier 1, 00133, Rome, Italy; federica.laudisi@gmail.com (F.L.); cherubini@med.uniroma2.it (F.C.); gi.monteleone@med.uniroma2.it (G.M.)

**Keywords:** STAT3, cancer, interactors, post-translational modifications, cytokines

## Abstract

Signal transducers and activators of transcription (STATs) mediate essential signaling pathways in different biological processes, including immune responses, hematopoiesis, and neurogenesis. Among the STAT members, STAT3 plays crucial roles in cell proliferation, survival, and differentiation. While STAT3 activation is transient in physiological conditions, STAT3 becomes persistently activated in a high percentage of solid and hematopoietic malignancies (e.g., melanoma, multiple myeloma, breast, prostate, ovarian, and colon cancers), thus contributing to malignant transformation and progression. This makes STAT3 an attractive therapeutic target for cancers. Initial strategies aimed at inhibiting STAT3 functions have focused on blocking the action of its activating kinases or sequestering its DNA binding ability. More recently, the diffusion of proteomic-based techniques, which have allowed for the identification and characterization of novel STAT3-interacting proteins able to modulate STAT3 activity via its subcellular localization, interact with upstream kinases, and recruit transcriptional machinery, has raised the possibility to target such cofactors to specifically restrain STAT3 oncogenic functions. In this article, we summarize the available data about the function of STAT3 interactors in malignant cells and discuss their role as potential therapeutic targets for cancer treatment.

## 1. Introduction

Intracellular signal transduction allows for the transmission of extracellular stimuli from the cell surface to a variety of intracellular targets to modulate the biological functions of a cell. Signal Transducers and Activators of Transcription (STATs) represent a family of latent cytoplasmic transcription factors involved in several pathways activated by both extrinsic and intrinsic signals that ultimately lead to the expression or repression of target genes involved in cell proliferation, apoptosis, inflammatory response, and angiogenesis [1].

The STAT family comprises seven members (i.e., STAT1, STAT2, STAT3, STAT4, STAT5α, STAT5β, and STAT6), which share conserved domains, such as the N-terminal coiled-coil domain, the DNA binding domain, a linker, the Src-homology 2 (SH2) domain, and the C-terminal transactivation domain [2]. Among the other STAT members, the transcription factor STAT3 is ubiquitously expressed and modulates genes involved in cell proliferation, survival, and differentiation [3]. While physiological STAT3 activation is transient, rapidly returning to the basal state, STAT3 becomes inappropriately and persistently activated in a wide variety of hematopoietic and solid malignancies, including melanoma, multiple myeloma, breast, prostate, ovarian, and colon cancer [4], due to upregulation of upstream signaling pathways attributed to molecules that are produced within the tumor microenvironment [5]. This hyperactivation promotes the expression of genes involved in cell proliferation (e.g., Cyclin D1, c-Myc), survival (e.g., Bcl-XL, survivin), immune suppression (e.g., IL-10), inflammation (e.g., COX-2, IL-6, IL-17A), and invasion and metastasis (e.g., vimentin, matrix metalloproteinases) [6] that altogether contribute to cancer initiation and progression.

Typically, STAT3 activation is triggered by the binding of cytokines and growth factors to their related receptors. For example, members of the IL-6 family of cytokines are potent activators of STAT3, mediating their signal through the pleiotropic gp130 receptor subunit that recruits and triggers the activation of the Janus kinase (JAK)2 by transphosphorylation [7]. JAK2 can then phosphorylate STAT3 on Tyr705 residue [8]. Phosphorylated STAT3 can homodimerize, as well as form dimers with STAT1, and then move into the nucleus to exert its functions [9]. It is noteworthy that STAT3 activation by tyrosine phosphorylation can be achieved also by several growth factors receptors (e.g., EGFR, HER2) and cytosolic kinases (e.g., Src, Abl) [10,11,12,13]. A second phosphorylation on Ser727, mediated by ERK and CDK1 among others [14,15], seems to enhance the transcriptional activity of Tyr705-phosphorylated STAT3 and enable STAT3 to have the capacity to regulate cell functions in the mitochondrion [16]. Within this organelle, STAT3 functions have been by and large correlated to Serine-phosphorylated STAT3. In particular, STAT3 modulates two major signalling pathways, the Electron Transport Chain (ETC) and the Mitochondrial Permeability Transition Pore (MPTP), influencing the mitochondrial membrane potential and proton gradient, ATP production, and reactive oxygen species (ROS) levels [17]. Mitochondrial STAT3 sustains altered glycolytic and oxidative phosphorylation activities characteristic of cancer cells, preventing cell death and favouring cell proliferation and metastasis. Thus, targeting STAT3’s mitochondrial function might be as important as targeting its transcriptional actions [17].

Besides tyrosine and serine phosphorylation, STAT3 functions in cancer cells can also be elicited by the fine tuning of other post-translational modifications (PTMs), such as methylation [18,19], ubiquitination [20], acetylation [21], SUMOylation [22], S-glutathionylation [23], and S-nitrosylation [24], through the modulation of STAT3 activity and/or stabilization/degradation. Conversely, the STAT3 signaling pathway can be inhibited by suppressor of cytokine signaling 3 (SOCS3), a STAT3-induced protein that is crucial for the proteasomal degradation of the gp130 receptor complex [25], as well as by SH2-containing phosphatases (e.g., SHP1, SHP2), that dephosphorylate JAK [26,27].

Targeting STAT3 is an appealing anti-cancer strategy and the multi-step process of STAT3 activation allows multiple points for inhibiting its oncogenic functions. Upstream kinases, such as JAKs, are among the STAT3 pathway components that have been targeted for cancer therapy. Such kinases tyrosine-phosphorylate STAT3, thus increasing its nuclear shuffling and transcriptional activity [28]. However, this approach is limited by the lack of a single STAT3 driver kinase in many cancer cells as well as by the development of resistance mutations in the targeted kinase or activation of other upstream kinases with redundant activities [10,11,12,13]. Moreover, accumulating evidence indicates that unphosphorylated STAT3 is also capable of dimerizing and inducing the transcription of genes which mostly overlap with those modulated by phosphorylated STAT3 [29]. Likewise, direct inhibition of STAT3 itself has proven challenging because of the high similarity with STAT1, a member mostly involved in cell death and defence against pathogens [30], and the lack of suitable docking pockets for the high-affinity binding of small molecules [31].

Because STAT3 mediates distinct biological effects by interacting with specific cooperating proteins, another strategy to inhibit oncogenic STAT3 functions might be the identification of tumor-specific STAT3 critical cofactors. In certain human cancers, the expression of critical oncogenes is driven by large regulatory elements, called super-enhancers, which are more densely bound chromatin regulators, transcriptional coactivators, and cofactors compared with their normal counterparts [32]. Thus, disrupting the structure of such super-enhancers or inhibiting the cofactors involved in the formation of super-enhancers represents a new and promising approach for cancer therapy [33,34]. Notably, STAT3 was recently reported to be one of the key transcription factors involved in the formation of super-enhancers [35,36] and this further pinpoints the role of STAT3 interactome as an exceptionally interesting drug target.

In this review, we summarize the role of STAT3-interacting proteins in malignant cells and discuss their potential as therapeutic targets for cancer treatment.

## 2. STAT3-Interacting Proteins

**Figure 1:**
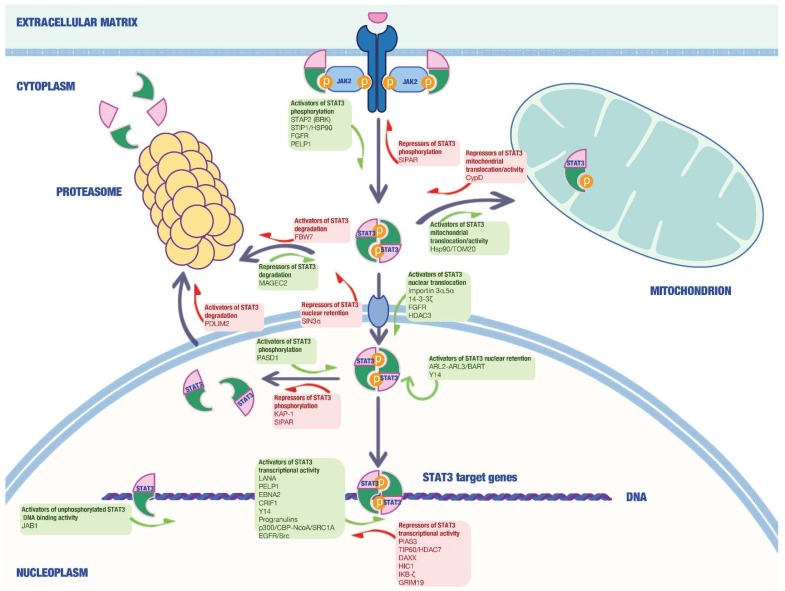
The figure illustrates localization and putative functions of the STAT3-interacting proteins discussed in this review. Green and red boxes enclose STAT3 activators and repressors respectively. Green and red arrows indicate positive and negative regulation of STAT3 expression/activity respectively.

In the last 20 years, several studies have identified and characterized a wide range of STAT3-interacting proteins in malignant cells able to modulate the activity of this transcription factor in a positive or negative manner in different cell compartments (Figure 1).

Such molecules, which can be considered, in all respects, potential valuable targets for cancer therapy, are summarized in Table 1 and Table 2 and discussed below.

### 2.1. STAT3 Activators

STAT3 interactors can positively regulate STAT3 activity in different cell compartments via a variety of mechanisms.

#### 2.1.1. STAT3 Activators in the Cytosol

In a study in 2003, Sato et al. described a role for Heat-shock protein 90 (Hsp90) in regulating IL-6 functions via STAT3 [37]. Hsp90 is highly expressed in the cytosol of eukaryotic cells and acts as a molecular chaperone for a number of signaling molecules [75]. Hsp90 directly interacted with STAT3 via its N-terminal region and the specific Hsp90 inhibitor geldanamycin suppressed IL-6-induced gene expression by interacting with STAT3 in the hepatoma cell line Hep3B. Consistently, Hsp90 overexpression reverted the inhibitory effects of geldanamycin on STAT3 activation [37]. As tumor cells are more dependent than normal cells on Hsp chaperons for proliferation and survival, this makes Hsp90 an attractive therapeutic target as suggested by accumulating evidence deriving from preclinical models and the clinic [76].

Stress-induced phosphoprotein 1 (STIP1) acts as an adapter molecule that directs HSP90 to HSP70–client protein complexes in the cytoplasm, thus modulating their chaperone activity [77]. High levels of STIP1 have been detected in a number of malignancies, including hepatocellular carcinoma, ovarian cancer, colon cancer, and pancreatic cancer [78,79,80,81]. In pancreatic cancer, Stip1 knockdown reduces tumor invasiveness via matrix metalloproteinase-2 downregulation [82]. The work of Tsai and colleagues demonstrated that STIP1 physically interacts with STAT3 in ovarian and endometrial cancer cells and is essential in the assembly and stabilization of the JAK2-HSP90-STAT3 complex, which promotes STAT3 phosphorylation and signal transduction [38]. Indeed, STIP1 knockdown by siRNA decreased JAK2 and phospho-STAT3 protein levels. Moreover, using truncated constructs of STIP1 and JAK2, the authors also showed that the N-terminal fragment of STIP1 binds with the N-terminus of JAK2, whereas the C-terminal DP2 domain of STIP1 mediates the interaction with STAT3 and HSP90. Consistently, a peptide fragment in the DP2 domain of STIP1 (peptide 520) disrupted the interaction between STIP1 and HSP90, thus affecting STAT3 signaling and leading to tumor growth inhibition in vitro and in vivo [38]. The above data suggest that repression of STIP1 may have therapeutic potential in the treatment of JAK2-overexpressing tumors.

The murine homolog of the human breast tumor kinase (BRK) substrate (BKS), also known as Signal transducing adaptor protein-2 (STAP-2), was seen to be a further critical STAT3-interacting protein playing an important role in BRK-dependent STAT3 activation [39]. STAP-2 belongs to the family of STAP adaptor proteins and it is composed by a pleckstrin homology (PH), SH2-like domain, and the YXXQ motif, a proline-rich region in the C-terminal domain which was reported to directly interact with STAT3 [83]. Ikeda and colleagues showed that BRK and STAP-2 are highly expressed in breast cancer cells and reported that STAP-2 is phosphorylated at tyrosine-250 by BRK, thus enhancing BRK-mediated STAT3 activation [39]. In a following study, the same authors expanded on such observations and, by manipulating STAP-2 expression, demonstrated that STAP-2 interacts with both BRK and STAT3 and plays essential roles in BRK-mediated STAT3 activation [84]. Indeed, silencing of endogenous STAP-2 expression by siRNA strongly reduced BRK-mediated STAT3 activation in T47D breast cancer cells. In addition, a STAP-2 PH-BRK fusion protein exhibited robust kinase activity and increased activation and tyrosine phosphorylation of STAT3. Consistently, STAP-2 knockdown in T47D cells significantly decreased cell proliferation, as strong as that of BRK or STAT3 knockdown [84]. Altogether, these observations highlight the crucial role of STAP-2 in BRK-mediated STAT3 activation and tumor cell growth and propose this molecule as a potential therapeutic target and prognostic factor for breast cancer patients.

The Fibroblast growth factor receptor (FGFR) family consists of Tyrosine Kinase Receptors involved in several biological functions and plays important roles for the progression and development of several cancers [85]. Using a proteomics approach, Dudka and colleagues identified STAT3 as a binding partner for phosphorylated Tyr677 of FGFR1 [40]. Enforced overexpression of FGFR1 in the human cervical carcinoma cell line HeLa induced STAT3 phosphorylation on tyrosine residues, nuclear translocation, and activation of its downstream target genes in contrast to cells with endogenous level of FGFRs. Tyrosine phosphorylation of STAT3 was also dependent on contemporary FGFR-dependent activity of JAK and SRC kinases. In addition, FGF stimulation of the FGFR2-overexpressing breast cancer cell line SUM-52PE also enhanced STAT3 tyrosine phosphorylation and transcriptional activity, which was strongly reduced by FGFR2 knockdown or pharmacologic inhibition [40]. Altogether, such findings indicate oncogenic overexpression of FGFR as an additional signaling pathway to induce aberrant STAT3 activation in tumor cells and suggest therapeutic strategies to treat FGFR-overexpressing cancers.

The 14-3-3 proteins are a family of molecules that interact with other proteins through serine/threonine-phosphorylated residues and function as adapter or scaffold proteins in different signaling pathways [86]. In particular, the isoform 14-3-3ζ has been identified as a prognostic marker and therapeutic target for multiple tumor types [87]. 14-3-3ζ protein interacts with the phosphorylated Ser727 of STAT3 in multiple myeloma cells and prevents the dephosphorylation of this critical residue by protein phosphatase 2A (PP2A). This results in an increased nuclear translocation, optimal DNA-binding, and transcriptional activity of STAT3 [41]. From a clinical perspective, however, it would be beneficial to target STAT3/14-3-3ζ interaction to inhibit the deleterious cooperation of 14-3-3ζ and STAT3 in cancer cells while sparing some of their essential homeostatic functions.

Cancer/testis antigens (CTAs) are tumor-associated antigens that are typically expressed only in adult testis but inappropriately reactivated in a variety of human cancers [88]. Song and colleagues explored the role of the CTA MAGEC2 in the process of cancer metastasis [42]. MAGEC2 was shown to interact with STAT3 and inhibit its polyubiquitination and proteasomal degradation in human and mouse melanoma cell lines (i.e., A375 and B16, respectively). This effect led to the accumulation of phosphorylated STAT3 and enhanced transcriptional activity that ultimately results in morphological changes of tumor cells and amoeboid migration. In vivo, intravenously injected MAGEC2-overexpressing B16 cells and MAGEC2-knockout A375 cells generated, respectively, more and less tumor nodules in the lungs of mice compared with parent cells. Such observations, together with evidence reporting a correlation between high levels of MAGEC2 and tumor metastasis in primary melanoma and breast cancer cells [89,90], indicate this protein as a promising target for treatment of tumor metastasis.

In order to bind the DNA and act as a transcription factor, STAT3 needs to translocate into the nucleus. Therefore, another possibility to modulate the STAT3 signaling pathway is to target proteins involved in STAT3 nuclear import. Although tyrosine phosphorylation is required for STAT3 to bind to specific DNA target sites, Liu and co-workers elegantly demonstrated that STAT3 nuclear import occurs constitutively through interaction with specific import carriers, namely importins, and independently of tyrosine phosphorylation [43]. In particular, they observed that importin α3 recognizes a constitutive nuclear localization sequence (NLS) in the coiled-coil domain of unphosphorylated STAT3 and that importin α3 silencing dramatically affects STAT3 nuclear localization in HeLa and Hep3B cells [43]. Such evidence differentiates STAT3 cellular localization from other STAT molecules and identifies a feature that could be targeted for clinical intervention in patients with STAT3-dependent cancers. Interestingly, Ha and Cao reported that other importins, such as importin α5 and importin α7, are also involved in STAT3 nuclear trafficking [44]. The importin α5 was described to bind tyrosine-phosphorylated STAT3 at the N-terminal domain through the specific binding site Arg214/215, as point mutations in this sequence were seen to affect STAT3 nuclear import [44]. The residues Arg414/417, instead, were crucial to maintain the appropriate conformation of STAT3 dimer for nuclear translocation. On the other hand, importin α7 was proposed to play a side role in STAT3 activation, establishing only a weak interaction with STAT3 [44].

#### 2.1.2. STAT3 Activators in the Nucleus

Consistently with the evidence that several tumor viruses are associated with STAT3 activation [91,92], Muromoto et al. reported that Kaposi’s sarcoma-associated herpesvirus (KSHV)-encoded latency-associated nuclear antigen (LANA) directly interacts with STAT3 and augments its transcriptional activation in primary effusion lymphoma (PEL) cell lines [45]. In particular, immunoprecipitation studies showed that LANA physically interacts with STAT3 through its C-terminal domain in KSHV-negative B lymphoma DG75 and KSHV-positive BC3 cells. LANA silencing by small interfering (si)RNA markedly reduced the expression of STAT3 target genes, such as cyclin D1 and CDK4, as well as the reporter gene expression from the STAT3–LUC construct compared to the control siRNA, without relevant change in the phosphorylation level of STAT3. Consistently, ectopically expressed LANA strongly increased STAT3–LUC levels in DG75 cells [45]. Later on, similar studies led to the identification of The Epstein–Barr virus (EBV)-encoded latency protein EBNA2 as a STAT3 interactor in HeLa cells [46]. Functional studies revealed that EBNA2 enhances STAT3 activity by augmenting its DNA-binding activity, thus acting as a transcriptional coactivator.

The same authors identified a new STAT3 activator, namely ADP-ribosylation factor like 2 (ARL2), which is able to mediate STAT3 transcriptional activity upon interaction with the binding partner binder of ARL2 (BART) [47]. In vitro assays in Hep3B and HeLa cells provided compelling evidence that BART protein is critical for STAT3 activation and nuclear retention following the interaction with ARL2. This high-affinity binding is GTP-dependent and results in the assembly of an ARL2-GTP-BART complex [47]. Some years later, BART was shown to interact also with another small GTP-binding protein, ADP-ribosylation factor like 3 (ARL3), which was proposed to be another critical STAT3-interacting protein. In particular, Togi and colleagues observed that ARL3 promotes STAT3 tyrosine phosphorylation and nuclear retention upon recognition of the STAT3 DNA-binding domain and C-terminal domain [48]. These data were further supported and confirmed by in vitro studies in HeLa cells, where reduction of ARL3 expression by siRNA resulted in less IL-6-induced STAT3 tyrosine phosphorylation as well as decreased STAT3 nuclear accumulation and transcriptional activity [48]. Moreover, consistently with the notion that ARL3 plays a role in microtubule-dependent processes [93], siRNA knockdown of ARL3 causes changes in cell morphology and reduced cell migration capacity [48].

Another important STAT3 interaction partner is represented by the RNA-binding protein Y14. In 2008, Ohbayashi et al. demonstrated that Y14 binds to STAT3 through the C-terminal region in the presence or absence of cytokine-induced STAT3 activation [49]. Knockdown experiments in Hep3B cells showed that Y14 is involved in tyrosine-phosphorylation, nuclear accumulation, and DNA-binding activity of STAT3. To clarify the physiological significance of the molecular interactions between STAT3 and Y14, the authors investigated the effect of Y14 on STAT3 activity. Small-interfering RNA-mediated lowering of Y14 expression significantly hampered IL-6/STAT3-dependent gene expression as indicated by the reduction of SOCS3 and C/EBPδ mRNA transcripts [49].

PAS domain containing 1 (PASD1) is a newly identified CTA demonstrated to interact with STAT3 in the nucleus by Xu and co-workers [50]. In HeLa cells, PASD1 overexpression enhanced both basal and IL-6-induced STAT3 activation, whereas knockdown of PASD1 had opposite effects. Mechanistically, PASD1 was seen to compete with TC45, a nuclear protein tyrosine phosphatase, for STAT3 binding, thus inhibiting TC45-mediated dephosphorylation of the transcription factor at Tyr705 residue. Consistently with this observation, knockdown of PASD1 inhibits the transcription of STAT3-related pro-oncogenic genes, leading to suppression of cell proliferation, anchorage-independent growth and cell migration in vitro, as well as the growth of HeLa-derived xenografts in nude mice [50].

Proline-, glutamic acid-, and leucine-rich protein-1 (PELP1) is an estrogen receptor coactivator identified as a STAT3-interacting protein by Manavathi et al. [51]. PELP1 overexpression in HeLa and MCF-7 cells enhanced STAT3 Ser727 phosphorylation in a Src-MAPK-sensitive manner, whereas downregulation of PELP1 hampered growth-factor-induced transcription of STAT3 target genes. In addition to this effect, PELP1 was shown to bind STAT3 in the nuclear compartment and facilitate its recruitment/retention in the target gene promoters [51]. As high levels of PELP1 have been seen in hormone-related cancers and associated with a poor prognosis [94], novel approaches aimed at selectively disrupting PELP1/STAT3 interaction may enable effective targeting of such malignancies.

Proteins interacting with STAT3 can modulate its activity independently of phosphorylation. In this context, a good example comes from Jun activation domain-binding protein 1 (JAB1). Nuclear JAB1 positively regulated unphosphorylated STAT3 DNA-binding activity through protein–protein interaction in the human colon cancer cell line Colo205 [52]. Conversely, JAB1 knockdown significantly reduced the RNA expression of STAT3 target genes (e.g., *MDR1*, *NANOG*, *VEGF*) [52]. Further investigation of the relationship between JAB1 and multiple oncogenic transcription factors containing STAT3 may enhance our knowledge of JAB1-mediated regulatory mechanisms and lead to the development of effective anti-cancer drugs.

Granulins are glycosylated peptides derived from the cleavage of the precursor protein progranulin (88 kDa), which is encoded by the *Grn* gene [95]. Granulin family members are involved in tumorigenesis and wound healing due to their modulatory effects on cell growth [96,97,98,99] Using a proteomic approach, Yeh and colleagues identified progranulin as a novel STAT3-interacting protein in triple-negative breast cancer (TNBC) cells bearing constitutively active STAT3 [53]. In such cells, progranulin silencing reduced STAT3 transcriptional activity by affecting both DNA binding and the recruitment of critical STAT3 cofactors. Functional experiments performed in SK-BR-3 cells showed that progranulin is necessary for maximal STAT3 transcriptional activity induced by multiple stimuli (i.e., leukemia inhibitory factor, IL-6, and oncostatin M). In addition, progranulin inhibition significantly hampered STAT3-mediated oncogenic phenotypes, such as clonogenesis and migratory capacity, in breast cancer cells. Consistently with the in vitro data, progranulin expression specifically correlated with enhanced STAT3 transcriptional activity in the presence of activated STAT3 in primary breast cancer samples and was associated with reduced overall survival in breast cancer patients [53].

CR6-interacting factor 1 (CRIF1), also known as GADD45GIP1, is a key regulator of cellular growth described to be involved in DNA damage and oxidative phosphorylation in the mitochondria [100,101]. Using the yeast two-hybrid assay, in 2008 Kwon et al. identified CRIF1 as a specific and essential transcriptional co-activator of STAT3 [54]. The authors found that CRIF1 recognizes the C-terminal coiled-coil domain of STAT3 protein, with consequent upregulation of STAT3 DNA-binding activity, whereas STAT3 phosphorylation or STAT3 nuclear translocation were not altered. In contrast to the strong interaction with STAT3, CRIF1 does not bind with STAT1 and STAT5a, suggesting a specific functional link between CRIF1 and STAT3. CRIF1 enhances the STAT3-mediated transcriptional activity in the presence or absence of leukemia inhibitory factor and oncostatin M in NIH-3T3 cells. Experiments performed in human colon, hepatic, and breast cancer cell lines (i.e., HCT-116, SNU387, and MDA-MB 468, respectively) indicated that the ectopic expression of CRIF1 without any stimulation greatly increases STAT3 transcriptional activity [54]. The constitutively active form of STAT3 (STAT3-C) was reported to induce cellular transformation of immortalized fibroblasts [102]. However, exogenous STAT3-C does not induce the cellular transformation of the immortalized CRIF1 conditionally inactivated mouse embryonic fibroblasts (MEFs), indicating that CRIF1 is indispensable for the STAT3-C-mediated oncogenic properties. Altogether, these results clearly show that CRIF1 inactivation completely blocks the transformation activity of STAT3. More recently, CRIF1 was seen to be highly expressed, together with STAT3, in the epithelium and stroma of prostate cancer and to compete with the coactivator p160 for the binding to the androgen receptor (AR), which is known to have a pro-tumorigenic role in this neoplasia [55]. This interaction results in the suppression of AR transcription activity and coactivation of STAT3. However, CRIF1 binding and co-activation of STAT3 may counteract the AR repressor effect of CRIF1, thus sustaining prostate cancer cell growth [55]. Therefore, inhibiting CRIF1 activity could be a novel therapeutic strategy to reduce oncogenic STAT3 functions in several types of cancer.

Besides its conventional role in promoting oncogenic responses through the ERK MAPK cascade and the activation of STAT family members [103], Epidermal Growth Factor Receptor (EGFR) has been detected in the cell nucleus and shown to function as a transcription factor through a non-traditional signaling mechanism [104]. Further investigation revealed that nuclear EGFR complexes with STAT3 in breast cancer cells induce the expression of specific genes, such as the inducible nitric oxide synthase (iNOS) [105]. On the same line is the work of Jaganathan et al., who presented evidence for a functional nuclear heteromeric EGFR, Src, and STAT3 complex in pancreatic cancer cells to promote the induction of the *c-Myc* gene [56]. Interestingly, cancer cells were more susceptible to concurrent modulation of any two of the EGFR, Src, and STAT3 proteins but not to the inhibition of the single proteins alone, thus suggesting the need for multiple-targeted therapy approaches for this deadly neoplasia.

STAT3 distribution and functional status in cancer cells can be modulated by acetylation. This makes components of the acetylation machinery possible therapeutic targets.

Yuan and coworkers demonstrated that STAT3 interacted with the Histone acetyltransferase (HAT)p300/CREB-binding protein (CBP) complex in Hela and MCF-7 cells and it was acetylated on a single lysine residue, Lys 685, in response to cytokine treatment [21]. STAT3 acetylation mediated by p300/CBP was attenuated by Type I histone deacetylase (HDAC)1 and 2 and almost completely inhibited by HDAC3 [21]. Using wild-type and mutant prostate cancer cell lines, the authors showed that Lys 685 acetylation was critical for STAT3 to form stable dimers, which are required for DNA binding and transcriptional regulation, in order to enhance transcription of cell-proliferation-related genes and promote cell cycle progression following cytokine stimulation [21].

Evidence of the crucial role of Lys 685 acetylation on STAT3 activity was given in parallel by another group in HepG2 cells [57]. In such cells, the steroid receptor co-activator 1 NcoA/SRC1a was shown to associate with p300/CBP following IL-6 stimulation and act as a cofactor to potentiate STAT3 transcriptional activity [58]. Pull-down experiments indicated that this ability relied on the association of the N-terminal part of NcoA/SRC1a with the activation domain of STAT3 and the CBP-interacting domain, activation domain 1 [58].

Gupta et al. uncovered the role of HDAC3 in regulating STAT3 activity in Diffuse large B-cell lymphoma (DLBCL) [59]. They showed that HDAC3 is aberrantly co-expressed with pSTAT3 Tyr705 in DLBCL tumor samples but not in normal blood B-cells and that HDAC3 complexed with STAT3 in pSTAT3 Tyr705-positive DLBCL cell lines (i.e., Ly3 and DHL2). Treatment of pSTAT3 DLBCL cells with the HDAC inhibitor panobinostat (LBH589) increased p300/CBP-mediated STAT3 Lys685 acetylation and abolished STAT3 Tyr705 phosphorylation with a negligible effect on STAT3 Ser727 and JAK2 tyrosine activity [59]. This effect was associated with decreased STAT3 protein in the nucleus and suggested that HDAC activity was required for cytokine-stimulated STAT3 nuclear translocation and transcriptional activity. Consistently, HDAC3 knockdown in Ly3 cells increased STAT3 Lys685 acetylation but prevented STAT3 Tyr705 phosphorylation with the ultimate result of increasing cell death [59]. These studies demonstrated the importance of HDAC3 in the modulation of STAT3 signaling in DLBCL cells and provided the rationale for targeting pSTAT3-positive DLBCL tumors with HDAC inhibitors. Currently, panobinostat is an approved drug for the treatment of lymphoma and multiple myeloma and a number of HDAC inhibitors are in clinical trials for the treatment of various malignancies [106].

#### 2.1.3. STAT3 Activators in the Mitochondrion

Besides its canonical function as transcription factor, STAT3 plays a distinct role in the mitochondria, where it supports Ras-dependent malignant transformation [107], promotes autophagy, and restrains ROS levels, thus increasing cancer cell survival [108].

Upstream activation by cytokines, growth factors, or oxidative stress phosphorylates STAT3 in Ser727, which seems crucial for STAT3 mitochondrial function [109]. Although not completely clear, the mitochondrial import of STAT3 relies on translocases of the mitochondrial outer membrane (TOM) complex, HSPs, and other chaperons [108]. Such components may thus represent possible therapeutic targets to hamper mitochondrial STAT3 oncogenic activity.

Mackenzie and coworkers demonstrated the importance of targeting mitochondrial STAT3 in pancreatic cancer [60]. They showed that the compound phospho-valproic acid (P-V) suppresses the interaction of STAT3 with Hsp90 and TOM20, both of which physically interact with STAT3, thus blocking STAT3 mitochondrial import [60]. This led to a depressed mitochondrial membrane potential, elevated ROS production, and increased mitochondrial-mediated apoptosis in pancreatic cancer cells. Mitochondrial levels of Hsp90 and Hsp60 proteins, both imported into the mitochondria, were not reduced by P-V, indicating that the variations in STAT3 levels did not rely on a generalized suppression of protein transport into the mitochondria. Importantly, the anti-cancer effect of P-V seemed to depend solely on inhibition of the actions of mitochondrial STAT3. In support of this hypothesis are results showing that human pancreatic cancer cell lines expressing a mitochondrial targeted STAT3, that were orthotopically implanted into nude mice, were completely insensitive to P-V-mediated growth inhibition [60].

### 2.2. STAT3 Repressors

Dephosphorylation and proteolytic degradation or impairment of DNA-binding ability have been shown to participate in the negative regulation of STAT3 functions [26].

While SOCS proteins interact with JAKs, thus reducing their tyrosine kinase activity, other inhibitors called PIAS (protein inhibitor of activated STAT) bind to activated STAT dimers and block their DNA binding activity [110]. The first evidence of a specific STAT3 repressor comes from the work of Chung and colleagues, who identified PIAS3 in 1997 [61]. The PIAS3 protein has been shown to bind specifically to activated STAT3 but not to other STATs and to inhibit STAT3-mediated gene activation in HepG2 and MCF-7 cells by blocking STAT3 DNA-binding activity [61]. In the following years, accumulating research pinpointed the role of this STAT3 suppressor as a promising therapeutic target in different cancer types [111,112].

Since then, several proteins have been identified as STAT3 interactors with negative effects on STAT3 functionality. One of the first-described STAT3 binding partners with repressive activity in cancer cells is TIP60 (Tat-interactive protein, 60 kDa), a protein involved in DNA damage repair, cell death apoptosis, histone acetylation, and the modulation of gene expression upon association with a number of transcription factors [113,114,115]. Xiao et al. showed that the C-terminal region of TIP60 associates with the DNA binding domain of STAT3 in HEK 293 cells and pointed out the ability of TIP60 to co-repress STAT3 activity in HepG2 in part upon recruitment of the histone deacetylase (HDAC)7 [62]. Interestingly, TIP60 overexpression could not repress the transcriptional activity of p53 or SMAD3, suggesting that TIP60 functions as a specific co-repressor for STAT3. Additionally, the authors also showed that ectopic expression of TIP60 in parental T lymphoma TS1 cells significantly attenuates the induction of the STAT3 target gene c-myc following IL-9 treatment. Altogether, these data indicate the ability of TIP60 to co-repress basal and cytokine-induced STAT3 activity in cancer cell lines in part through the recruitment of HDAC7.

Type I interferon (IFN) induces the expression of pro-apoptotic genes and has been used in the clinical treatment of a number of tumors [116,117,118,119,120]. The Type I-IFN-induced Death domain-associated protein (DAXX) is a potent transcription repressor known to interact with several transcription factors (e.g., ETS1, RelA, RelB, SMAD4) and modulate their activities [121,122,123,124]. In 2006, Muromoto and colleagues demonstrated that DAXX interacts with nuclear STAT3 and reduces its transcriptional activity in response to type I IFN signaling [63]. Indeed, pre-treatment of HeLa and Hep3B cells with IFN decreased IL-6-induced STAT3 transcriptional activities. Further studies revealed that the N-terminal domain of DAXX interacts with the DNA-binding domain of STAT3, thus affecting STAT3-binding to the consensus DNA sequence [63]. Importantly, IL-6-mediated endogenous SOCS3 mRNA expression was markedly decreased in DAXX-overexpressing, but not in parental, Hep3B cells, suggesting that DAXX negatively regulates IL-6/STAT3-mediated gene expression. On the other hand, reduction of endogenous DAXX expression in HeLa cells via siRNA resulted in a strong increase of cytokine-induced STAT3 activation and SOCS3 mRNA expression [63]. Two years later, the same group identified another important STAT3 binding protein with suppressive effects on STAT3 functions, namely KRAB-associated protein-1 (KAP-1) [64]. Co-immunoprecipitation experiments showed that KAP1 physically interacts with STAT3 in Hep3B cells. In such cells, KAP1 knockdown by specific siRNA significantly enhances STAT3 activation as well as the expression of STAT3-related RNA transcripts (e.g., SOCS3 c-myc, Mcl-1) in the presence or absence of IL-6 stimulation [64]. Mechanistically, reduction of KAP1 expression in Hep3B cells does not affect STAT3 nuclear translocation and DNA-binding activity, but resulted in enhanced nuclear accumulation of STAT3 phosphorylated on Ser727, a modification required for its maximal transcriptional activation [125]. STAT3 activation can also be regulated via the ubiquitin/proteasome-dependent degradation of STAT3 [69,126,127]. Tanaka et al. reported that PDLIM2, a nuclear ubiquitin E3 ligase, binds to and degrades STAT3 in a proteasome-dependent manner in human embryonic kidney (HEK) 293T cells [65]. Consistently, in Hep3B cells, specific knockdown of PDLIM2 by siRNA causes insufficient STAT3 degradation, resulting in a nuclear accumulation of STAT3 and enhanced STAT3 transactivation induced by IL-6, thus supporting a role for PDLIM2 in inhibiting both the amount of STAT3 protein and the extent of STAT3 activation [65]. Together with PDLIM2, IκB-ζ was also found to be a negative regulator of STAT3 activity [66]. IκB-ζ is a member of the IκB family, which has been described to be an important regulator of Nuclear factor kappa-light-chain-enhancer of activated B cells (NF-κB) transcription factor and whose activity is induced in immune cells upon stimulation with inflammatory cytokines (e.g., IL-1β, TNF-α) and microbial Lipopolysaccharide [128,129]. By a co-immunoprecipitation and yeast two-hybrid screening assay, Wu and co-workers clearly revealed that IκB-ζ binds the STAT3 coiled-coil domain via its C-terminal domain, exerting a negative effect on STAT3 function [66]. In HeLa cells, this interaction is responsible for the arrest of cell growth and induction of apoptosis, due in part to the downregulation of the STAT3 target gene *Mcl-1* [66].

Hypermethylated in cancer 1 (HIC1) is a tumor suppressor gene that is frequently deleted or epigenetically silenced in a variety of human cancers [130]. STAT3 was identified as a HIC1-interacting protein by Lin et al. [68]. In the breast cancer cell line MDA-MB-231, overexpression or depletion of HIC1 resulted in decreased or increased levels of cytokine-induced STAT3-target gene RNA transcripts. Moreover, the effect of HIC1 on cell growth inhibition was dampened in STAT3-lacking cells, suggesting the crucial role of HIC1 in counteracting STAT3-mediated cell growth. Mechanistically, HIC1 interacts with the DNA-binding domain of STAT3 via its C-terminal domain, thus suppressing the binding of STAT3 to its target gene promoters [68]. More recently, Hu and colleagues confirmed HIC1’s repressor activity on STAT3 in pancreatic cancer [67]. HIC1 expression was reduced in pancreatic cancer cell lines and in tumor tissues of pancreatic cancer patients compared with non-transformed cells. Negative HIC1 expression predicted advanced pathological stages of the disease and worse patient prognosis and survival. Notably, inhibition of HIC1-mediated STAT3–DNA binding affected invasion and metastasis of pancreatic cancer cells both in vitro and in vivo [67]. As loss of HIC1 expression is observed in a large number of tumors and associated with enhanced STAT3 activity and tumorigenesis. Increasing HIC1 expression and/or HIC1–STAT3 interaction could represent a new and promising approach to treat STAT3-associated human cancers.

The ubiquitin-ligase Fbw7 acts as a tumor suppressor, tagging a large number of proto-oncogenes for proteasomal degradation [131]. Recently, Yao and coauthors reported a tight interaction of Fbw7 and STAT3 in activated B-cell-like diffuse large B cell lymphoma (ABC-DLBCL) [69]. Ectopic expression of Fbw7 in the ABC-DLBCL cell lines SU-DHL-2 and OCI-LY-3 led to STAT3 and pSTAT3 Tyr705 stability reduction, decreased cell viability, and increased apoptosis rates. Accordingly, STAT3 target genes, which mediate anti-apoptotic effects (e.g., Myc, Survivin, Bcl-2), were downregulated following Fbw7 expression in ABC-DLBCL cells [69]. The negative correlation between Fbw7 and STAT3 activity was confirmed in DLBCL patient samples [69]. Such results indicate that Fbw7 targets STAT3 for ubiquitylation and degradation to regulate apoptosis in ABC-DLBCL, thus suggesting a new method of STAT3 inhibition for cancer therapy.

In 2013, Chang’s group identified a novel gene encoding a protein named STAT3 Interacting Protein as a Repressor (SIPAR) [70]. They observed that SIPAR physically interacts with STAT3 in melanoma cells and inhibits STAT3 activity by accelerating its dephosphorylation. Overexpression of SIPAR in the murine melanoma cell line B16 inhibited the expression of STAT3 target genes and cell growth in vitro and repressed melanoma progression in nude mice [70]. In a following study, the same group demonstrated that SIPAR reduced STAT3 activity by enhancing the interaction of STAT3 with the tyrosine phosphatase TC45 [132].

Icardi et al. uncovered the role of Sin3 transcription regulator homolog A (Sin3a) as a repressor of STAT3 transcriptional activity [71]. Sin3a directly interacted with STAT3 to modify its acetylation pattern and nucleocytoplasmic distribution. Moreover, SIN3a silencing in MCF-7 and HepG2 cells resulted in hyperacetylation and prolonged nuclear retention of activated STAT3, leading to an increased transcription of STAT3-target genes [71]. From a clinical perspective, high levels of Sin3a have been shown to correlate with longer relapse-free survival in patients with triple-negative breast cancer [133]. However, therapeutic approaches aimed at targeting Sin3a should take into account that this molecule is engaged in multiple protein–protein interactions and has multiple paralogs with overlapping functions. Modulating Sin3a levels can have a different effect on the function of the residual complex rather than interfering only with specific protein interactions of Sin3a [134].

Zhang et al. identified GRIM-19 (gene associated with retinoid-IFN-induced mortality-19) as a specific STAT3 binding partner in different breast cancer cell lines (i.e., MCF-7, T47D, and BT20) [72]. Functional experiments in MCF-7 cells highlighted an exclusive negative regulatory effect of GRIM-19 on STAT3- but not STAT1-dependent gene expression. Mechanistically, mutational analysis indicated that the transactivation domain of STAT3, especially residue Ser727, is required for GRIM-19 binding to STAT3. Moreover, the authors elegantly showed that the repressor function of GRIM-19 on STAT3 is not due to changes in STAT3 phosphorylation status, as no decrease in tyrosine or serine of STAT3 is observed in GRIM-19-expressing cells, and that neither the DNA binding nor the ligand-induced nuclear translocation of STAT3 are inhibited in the presence of GRIM-19 [72]. GRIM-19 is also a component of complex I in the electron transport chain and acts as a chaperone to recruit STAT3 into the inner mitochondrial membrane, thus modulating its mitochondria-specific functions [135]. Recently, Huang and colleagues suggested mitochondrial GRIM-19 as potential therapeutic target for STAT3-dependent carcinogenesis of gastric cancer [73].

Mitochondrial STAT3 also interacts with Cyclophilin D (CypD) to regulate the mitochondrial permeability transition pore (MPTP) [136]. Deletion or decreased expression of mitochondrial CypD activated interorganelle signaling in glioblastoma and breast cancer cell lines, leading to transcriptional changes in gene expression, modulation of a chemokine/chemokine receptor signature, and STAT3 activation [74]. In turn, this favored cell cycle progression through the S-phase and enhanced chemokine-dependent autocrine/paracrine cell migration and invasion [74].

## 3. Conclusions

The transcription factor STAT3 is activated downstream of cytokines and growth factors to elicit different and sometimes contrasting effects under both physiological and pathological conditions. Inappropriate/persistent STAT3 activation is reported in a variety of clinical tumor samples, where it seems to drive multiple pro-oncogenic functions. Consistently, blockade of STAT3 in cultured cancer cells was found to inhibit cell proliferation, induce apoptosis, and stimulate immune responses. For that, STAT3 is widely considered as an oncogene and an attractive therapeutic target, although recent evidence have proved its ability to suppress malignant cell onset and/or progression in particular tumor backgrounds [137]. Thus far, common approaches aimed at inhibiting STAT3 functions by direct targeting or blocking the function of its activating kinases have been challenged by several limitations. Also, inhibiting STAT3 at the source of activating signaling pathways would be deleterious because normal cells can also be affected.

In recent years, proteomics-based approaches have shed light on STAT3 interactors and their effects on physiologic and neoplastic STAT3 functions. In particular, analysis of STAT3-containing complexes has allowed us to identify novel STAT3-binding proteins which can modulate the oncogenic activity of the transcription factor via its subcellular localization, DNA binding ability, and recruitment of transcriptional machinery. Outlining the role of such proteins might increase our knowledge about the functional regulation of STAT3 and reveal targetable elements in its signaling pathway that have previously been undervalued. This may allow a tumor-cell- and tissue-specific targeting of STAT3 while sparing healthy cells. Moreover, a comprehensive characterization of the STAT3 structural domains involved in these protein–protein interactions would permit a more selective inhibition of a cohort of STAT3 target genes with oncogenic but not physiologic functions in order to restrain therapy-related side effects.

Like STAT3, the transcription factor NF-κB is aberrantly activated in many types of cancers, where it regulates, among others, genes involved in tumor cell proliferation, migration, and invasion [138]. Importantly, STAT3 is known to directly bind, through its DNA-binding domain, to the transactivation domain of NF-κB [139], and this interaction plays a key role in controlling the dialog between the malignant cell and its microenvironment in part through the induction of certain gene subsets that require cooperation between the two transcription factors [5]. Of note is that some of the STAT3-binding proteins indicated in this review also directly interact with NF-κB [123,140,141,142,143]. Thus, targeting such molecules, besides the effect on STAT3-regulated genes, could help inhibit STAT3/NF-κB interaction/cross-talk and the related oncogenic activities.

In conclusion, therapeutic strategies aimed at targeting specific STAT3 interactors are likely to have potential to restrain STAT3-mediated cancer progression with limited detrimental effects on normal cells. However, studies that demonstrate their anti-tumor efficacy and lack of toxicity in relevant preclinical animal models of human cancer are lacking and urgently needed in order to better understand the feasibility of such a therapeutic approach in cancer patients.

## Figures and Tables

**Table 1:** STAT3 activators.

Name	Mechanism	Cancer Type	Ref.
Hsp90	Chaperone activity via interaction with STAT3 N-terminal region	Hep3B cells	[37]
STIP1	Stabilization of JAK2 through its N-terminal domain, thereby promoting STAT3 phosphorylation, signal transduction and JAK2-HSP90-STAT3 complex assembly	MDAH2774, SKOV3 and ARK2 cells, primary ovarian tissues	[38]
STAP-2	Modulation of STAT3 activity upon phosphorylation by BRK	HeLa, MCF-7 and T47D cells	[39]
FGFR	Induction of STAT3 phosphorylation on tyrosine residues, nuclear translocation and activation of STAT3 target genes	HeLa and SUM-52PE cells	[40]
14-3-3ζ	Interacts with and prevents pSTAT3 Ser727 dephosphorylation by PP2A phosphatase	Multiple myeloma cells	[41]
MAGEC2	Interacts with and inhibits STAT3 polyubiquitination and proteasomal degradation	Human (A375) and mouse (B16) melanoma cell lines	[42]
Importin 3α	Binding to the nuclear localization sequence in the coiled-coil domain of unphosphorylated STAT3	HeLa and Hep3B cells	[43]
Importin 5α	Binding to tyrosine phosphorylated STAT3 at the N-terminal domain and maintenance of appropriate conformation	HepG2, MCF7 and HeLa cells	[44]
LANA	Interaction with STAT3 C-terminal domain and increased STAT3 transcriptional activity	KSHV-negative B lymphoma, DG75 and KSHV-positive BC3 cells	[45]
EBNA2	Increased STAT3 DNA-binding activity	HeLa cells	[46]
ARL2/BART	ARL2-GTP-BART complex is critical for STAT3 activation and nuclear translocation	Hep3B and HeLa cells	[47]
ARL3	Interaction with STAT3 DNA-binding domain and C-terminal domain, with consequent STAT3 phosphorylation	HeLa cells	[48]
Y14	Association with STAT3 C-terminal domain and positive regulation of STAT3 function	Hep3B cells	[49]
PASD1	Impairment of nuclear STAT3 dephosphorylation by TC45	HeLa cells, HeLa-derived xenografts in nude mice	[50]
PELP1	Increases STAT3 phosphorylation on Ser727 residue, facilitates STAT3 recruitment/retention in the target gene promoters	HeLa and MCF-7 cells	[51]
JAB1	Increases unphosphorylated STAT3 DNA-binding activity	Colo205 colon cancer cells	[52]
Progranulin	Regulation of STAT3 phosphorylation, nuclear translocation and transcriptional activity	TNBC cell lines. Primary breast cancer samples	[53]
CRIF1	Association with STAT3 C-terminal coiled-coil domain and positive regulation of STAT3 transcription activity	HeLa, HCT-116, SNU387 and MDA-MB 468 cells	[54]
	Binding and suppression of the androgen receptor transcription activity and coactivation of STAT3	CWR-R1 cells and surgical specimens of prostate	[55]
EGFR	Acts as a transcriptional co-activator of STAT3	Panc-1 and Colo-357 pancreatic cancer cell lines	[56]
p300/CREB	Regulation of Lys 685 acetylation, critical for STAT3 to form stable dimers and required for DNA binding	HeLa, MCF-7 and HepG2 cells	[21,57]
NcoA/SRC1a	Associates with p300/CBP and acts as a cofactor to potentiate STAT3 transcriptional activity	HepG2 cells	[58]
HDAC3	Modulation of STAT3 Lys685 acetylation and Tyr705 phosphorylation	Ly3 and DHL2 DLBCL cell lines	[59]
TOM20	Regulates STAT3 mitochondrial import and oncogenic functions	Human pancreatic cell lines. BxPC-3-and MIA PaCa-2-derived xenografts in nude mice	[60]

TNBC = Triple-Negative Breast Cancer; DLBCL = Diffuse Large B-Cell Lymphoma.

**Table 2:** STAT3 repressors.

Name	Mechanism	Cancer Type	Ref.
PIAS3	Inhibition of STAT3-mediated gene activation	HepG2 and MCF-7 cells	[61]
TIP60	Repression of STAT3 activity upon HDAC7 recruitment with its central domain	HepG2 and TS1 cells	[62]
DAXX	Binding and down-regulation of nuclear STAT3 in response to type I IFN signaling by impairing STAT3-binding to the consensus DNA sequence	HeLa and Hep3B cells	[63]
KAP-1	Impairment of STAT3 phosphorylation status on Ser727 residue by competing with p300	Hep3B cells	[64]
PDLIM2	Degradation of STAT3 in a proteasome-dependent manner	Hep3B cells	[65]
IKB-ζ	Binding to STAT3 coil-coiled domain	HeLa cells	[66]
HIC1	Interacts with the DNA binding domain of STAT3 via its C-terminal domain thus suppressing the binding of STAT3 to its target gene promoters	MDA-MB-231 breast cancer cells, pancreatic cancer cell lines	[67,68]
Fbw7	STAT3 and pSTAT3 Tyr705 stability reduction	ABC-DLBCL cell lines SU-DHL-2 and OCI-LY-3	[69]
SIPAR	Accelerates STAT3 dephosphorylation by enhancing the interaction of STAT3 with the tyrosine phosphatase TC45	B16 mouse melanoma cells	[70]
Sin3A	Modulates STAT3 acetylation pattern and nucleocytoplasmic distribution	HepG2 and MCF-7 cells	[71]
GRIM-19	Binding to Ser727 residue on STAT3 and inhibition of STAT3-mediated gene expression	MCF-7, T47D and BT-20 cells	[72]
	Attenuation of STAT3 nuclear translocation	SGC-7901 and BGC-823 gastric cancer cells	[73]
CypD	Interacts with mitochondrial STAT3 to regulate the MPTP	Glioblastoma and breast cancer cell lines	[74]

ABC-DLBCL = Activate B Cell like-Diffuse Large B-Cell Lymphoma; MPTP = Mitochondrial Permeability Transition Pore.

## References

[1] Abroun S., Saki N., Ahmadvand M., Asghari F., Salari F., Rahim F. (2015). STATs: An Old Story, Yet Mesmerizing. Cell J..

[2] Miklossy G., Hilliard T.S., Turkson J. (2013). Therapeutic modulators of STAT signalling for human diseases. Nat. Rev. Drug Discov..

[3] Demaria M., Camporeale A., Poli V. (2014). STAT3 and metabolism: How many ways to use a single molecule?. Int. J. Cancer.

[4] Bowman T., Garcia R., Turkson J., Jove R. (2000). STATs in oncogenesis. Oncogene.

[5] Grivennikov S.I., Karin M. (2010). Dangerous liaisons: STAT3 and NF-kappaB collaboration and crosstalk in cancer. Cytokine Growth Factor Rev..

[6] Carpenter R.L., Lo H.W. (2014). STAT3 Target Genes Relevant to Human Cancers. Cancers.

[7] Garbers C., Aparicio-Siegmund S., Rose-John S. (2015). The IL-6/gp130/STAT3 signaling axis: Recent advances towards specific inhibition. Curr. Opin. Immunol..

[8] Rawlings J.S., Rosler K.M., Harrison D.A. (2004). The JAK/STAT signaling pathway. J. Cell Sci..

[9] Bromberg J., Darnell J.E. (2000). The role of STATs in transcriptional control and their impact on cellular function. Oncogene.

[10] Turkson J., Bowman T., Garcia R., Caldenhoven E., De Groot R.P., Jove R. (1998). Stat3 activation by Src induces specific gene regulation and is required for cell transformation. Mol. Cell. Biol..

[11] Coppo P., Dusanter-Fourt I., Millot G., Nogueira M.M., Dugray A., Bonnet M.L., Mitjavila-Garcia M.T., Le Pesteur D., Guilhot F., Vainchenker W. (2003). Constitutive and specific activation of STAT3 by BCR-ABL in embryonic stem cells. Oncogene.

[12] Sriuranpong V., Park J.I., Amornphimoltham P., Patel V., Nelkin B.D., Gutkind J.S. (2003). Epidermal growth factor receptor-independent constitutive activation of STAT3 in head and neck squamous cell carcinoma is mediated by the autocrine/paracrine stimulation of the interleukin 6/gp130 cytokine system. Cancer Res..

[13] Coppo P., Flamant S., De Mas V., Jarrier P., Guillier M., Bonnet M.L., Lacout C., Guilhot F., Vainchenker W., Turhan A.G. (2006). BCR-ABL activates STAT3 via JAK and MEK pathways in human cells. Br. J. Haematol..

[14] Andres R.M., Hald A., Johansen C., Kragballe K., Iversen L. (2013). Studies of Jak/STAT3 expression and signalling in psoriasis identifies STAT3-Ser727 phosphorylation as a modulator of transcriptional activity. Exp. Dermatol..

[15] Shi X., Zhang H., Paddon H., Lee G., Cao X., Pelech S. (2006). Phosphorylation of STAT3 serine-727 by cyclin-dependent kinase 1 is critical for nocodazole-induced mitotic arrest. Biochemistry.

[16] Yang R., Rincon M. (2016). Mitochondrial Stat3, the Need for Design Thinking. Int. J. Biol. Sci..

[17] Meier J.A., Larner A.C. (2014). Toward a new STATe: The role of STATs in mitochondrial function. Semin. Immunol..

[18] Yang J., Huang J., Dasgupta M., Sears N., Miyagi M., Wang B., Chance M.R., Chen X., Du Y., Wang Y. (2010). Reversible methylation of promoter-bound STAT3 by histone-modifying enzymes. Proc. Natl. Acad. Sci. USA.

[19] Dasgupta M., Dermawan J.K., Willard B., Stark G.R. (2015). STAT3-driven transcription depends upon the dimethylation of K49 by EZH2. Proc. Natl. Acad. Sci. USA.

[20] Perry E., Tsruya R., Levitsky P., Pomp O., Taller M., Weisberg S., Parris W., Kulkarni S., Malovani H., Pawson T. (2004). TMF/ARA160 is a BC-box-containing protein that mediates the degradation of Stat3. Oncogene.

[21] Yuan Z.L., Guan Y.J., Chatterjee D., Chin Y.E. (2005). Stat3 dimerization regulated by reversible acetylation of a single lysine residue. Science.

[22] Zhou Z., Wang M., Li J., Xiao M., Chin Y.E., Cheng J., Yeh E.T., Yang J., Yi J. (2016). SUMOylation and SENP3 regulate STAT3 activation in head and neck cancer. Oncogene.

[23] Butturini E., Darra E., Chiavegato G., Cellini B., Cozzolino F., Monti M., Pucci P., Dell’Orco D., Mariotto S. (2014). S-Glutathionylation at Cys328 and Cys542 impairs STAT3 phosphorylation. ACS Chem. Biol..

[24] Kim J., Won J.S., Singh A.K., Sharma A.K., Singh I. (2014). STAT3 regulation by S-nitrosylation: Implication for inflammatory disease. Antioxid. Redox Signal..

[25] Suzuki A., Hanada T., Mitsuyama K., Yoshida T., Kamizono S., Hoshino T., Kubo M., Yamashita A., Okabe M., Takeda K. (2001). CIS3/SOCS3/SSI3 plays a negative regulatory role in STAT3 activation and intestinal inflammation. J. Exp. Med..

[26] Shuai K., Liu B. (2003). Regulation of JAK-STAT signalling in the immune system. Nat. Rev. Immunol..

[27] Yasukawa H., Sasaki A., Yoshimura A. (2000). Negative regulation of cytokine signaling pathways. Annu. Rev. Immunol..

[28] Levy D.E., Lee C.K. (2002). What does Stat3 do?. J. Clin. Investig..

[29] Yang J., Chatterjee-Kishore M., Staugaitis S.M., Nguyen H., Schlessinger K., Levy D.E., Stark G.R. (2005). Novel roles of unphosphorylated STAT3 in oncogenesis and transcriptional regulation. Cancer Res..

[30] Avalle L., Pensa S., Regis G., Novelli F., Poli V. (2012). STAT1 and STAT3 in tumorigenesis: A matter of balance. Jak-Stat.

[31] Nero T.L., Morton C.J., Holien J.K., Wielens J., Parker M.W. (2014). Oncogenic protein interfaces: Small molecules, big challenges. Nat. Rev. Cancer.

[32] Pott S., Lieb J.D. (2015). What are super-enhancers?. Nat. Genet..

[33] Loven J., Hoke H.A., Lin C.Y., Lau A., Orlando D.A., Vakoc C.R., Bradner J.E., Lee T.I., Young R.A. (2013). Selective inhibition of tumor oncogenes by disruption of super-enhancers. Cell.

[34] Hnisz D., Schuijers J., Lin C.Y., Weintraub A.S., Abraham B.J., Lee T.I., Bradner J.E., Young R.A. (2015). Convergence of developmental and oncogenic signaling pathways at transcriptional super-enhancers. Mol. Cell.

[35] Hnisz D., Abraham B.J., Lee T.I., Lau A., Saint-Andre V., Sigova A.A., Hoke H.A., Young R.A. (2013). Super-enhancers in the control of cell identity and disease. Cell.

[36] Li P., Mitra S., Spolski R., Oh J., Liao W., Tang Z., Mo F., Li X., West E.E., Gromer D. (2017). STAT5-mediated chromatin interactions in superenhancers activate IL-2 highly inducible genes: Functional dissection of the Il2ra gene locus. Proc. Natl. Acad. Sci. USA.

[37] Sato N., Yamamoto T., Sekine Y., Yumioka T., Junicho A., Fuse H., Matsuda T. (2003). Involvement of heat-shock protein 90 in the interleukin-6-mediated signaling pathway through STAT3. Biochem. Biophys. Res. Commun..

[38] Tsai C.L., Chao A., Jung S.M., Tsai C.N., Lin C.Y., Chen S.H., Sue S.C., Wang T.H., Wang H.S., Lai C.H. (2016). Stress-induced phosphoprotein-1 maintains the stability of JAK2 in cancer cells. Oncotarget.

[39] Ikeda O., Miyasaka Y., Sekine Y., Mizushima A., Muromoto R., Nanbo A., Yoshimura A., Matsuda T. (2009). STAP-2 is phosphorylated at tyrosine-250 by Brk and modulates Brk-mediated STAT3 activation. Biochem. Biophys. Res. Commun..

[40] Dudka A.A., Sweet S.M., Heath J.K. (2010). Signal transducers and activators of transcription-3 binding to the fibroblast growth factor receptor is activated by receptor amplification. Cancer Res..

[41] Zhang J., Chen F., Li W., Xiong Q., Yang M., Zheng P., Li C., Pei J., Ge F. (2012). 14-3-3zeta interacts with stat3 and regulates its constitutive activation in multiple myeloma cells. PLoS ONE.

[42] Song X., Hao J., Wang J., Guo C., Wang Y., He Q., Tang H., Qin X., Li Y., Zhang Y. (2017). The cancer/testis antigen MAGEC2 promotes amoeboid invasion of tumor cells by enhancing STAT3 signaling. Oncogene.

[43] Liu L., McBride K.M., Reich N.C. (2005). STAT3 nuclear import is independent of tyrosine phosphorylation and mediated by importin-alpha3. Proc. Natl. Acad. Sci. USA.

[44] Ma J., Cao X. (2006). Regulation of Stat3 nuclear import by importin alpha5 and importin alpha7 via two different functional sequence elements. Cell. Signal..

[45] Muromoto R., Okabe K., Fujimuro M., Sugiyama K., Yokosawa H., Seya T., Matsuda T. (2006). Physical and functional interactions between STAT3 and Kaposi’s sarcoma-associated herpesvirus-encoded LANA. FEBS Lett..

[46] Muromoto R., Ikeda O., Okabe K., Togi S., Kamitani S., Fujimuro M., Harada S., Oritani K., Matsuda T. (2009). Epstein-Barr virus-derived EBNA2 regulates STAT3 activation. Biochem. Biophys. Res. Commun..

[47] Muromoto R., Sekine Y., Imoto S., Ikeda O., Okayama T., Sato N., Matsuda T. (2008). BART is essential for nuclear retention of STAT3. Int. Immunol..

[48] Togi S., Muromoto R., Hirashima K., Kitai Y., Okayama T., Ikeda O., Matsumoto N., Kon S., Sekine Y., Oritani K. (2016). A New STAT3-binding Partner, ARL3, Enhances the Phosphorylation and Nuclear Accumulation of STAT3. J. Biol. Chem..

[49] Ohbayashi N., Taira N., Kawakami S., Togi S., Sato N., Ikeda O., Kamitani S., Muromoto R., Sekine Y., Matsuda T. (2008). An RNA biding protein, Y14 interacts with and modulates STAT3 activation. Biochem. Biophys. Res. Commun..

[50] Xu Z.S., Zhang H.X., Zhang Y.L., Liu T.T., Ran Y., Chen L.T., Wang Y.Y., Shu H.B. (2016). PASD1 promotes STAT3 activity and tumor growth by inhibiting TC45-mediated dephosphorylation of STAT3 in the nucleus. J. Mol. Cell Biol..

[51] Manavathi B., Nair S.S., Wang R.A., Kumar R., Vadlamudi R.K. (2005). Proline-, glutamic acid-, and leucine-rich protein-1 is essential in growth factor regulation of signal transducers and activators of transcription 3 activation. Cancer Res..

[52] Nishimoto A., Kugimiya N., Hosoyama T., Enoki T., Li T.S., Hamano K. (2013). JAB1 regulates unphosphorylated STAT3 DNA-binding activity through protein-protein interaction in human colon cancer cells. Biochem. Biophys. Res. Commun..

[53] Yeh J.E., Kreimer S., Walker S.R., Emori M.M., Krystal H., Richardson A., Ivanov A.R., Frank D.A. (2015). Granulin, a novel STAT3-interacting protein, enhances STAT3 transcriptional function and correlates with poorer prognosis in breast cancer. Genes Cancer.

[54] Kwon M.C., Koo B.K., Moon J.S., Kim Y.Y., Park K.C., Kim N.S., Kwon M.Y., Kong M.P., Yoon K.J., Im S.K. (2008). Crif1 is a novel transcriptional coactivator of STAT3. EMBO J..

[55] Tan J.A., Bai S., Grossman G., Titus M.A., Harris Ford O., Pop E.A., Smith G.J., Mohler J.L., Wilson E.M., French F.S. (2014). Mechanism of androgen receptor corepression by CKbetaBP2/CRIF1, a multifunctional transcription factor coregulator expressed in prostate cancer. Mol. Cell. Endocrinol..

[56] Jaganathan S., Yue P., Paladino D.C., Bogdanovic J., Huo Q., Turkson J. (2011). A functional nuclear epidermal growth factor receptor, SRC and Stat3 heteromeric complex in pancreatic cancer cells. PLoS ONE.

[57] Wang R., Cherukuri P., Luo J. (2005). Activation of Stat3 sequence-specific DNA binding and transcription by p300/CREB-binding protein-mediated acetylation. J. Biol. Chem..

[58] Giraud S., Bienvenu F., Avril S., Gascan H., Heery D.M., Coqueret O. (2002). Functional interaction of STAT3 transcription factor with the coactivator NcoA/SRC1a. J. Biol. Chem..

[59] Gupta M., Han J.J., Stenson M., Wellik L., Witzig T.E. (2012). Regulation of STAT3 by histone deacetylase-3 in diffuse large B-cell lymphoma: Implications for therapy. Leukemia.

[60] Mackenzie G.G., Huang L., Alston N., Ouyang N., Vrankova K., Mattheolabakis G., Constantinides P.P., Rigas B. (2013). Targeting mitochondrial STAT3 with the novel phospho-valproic acid (MDC-1112) inhibits pancreatic cancer growth in mice. PLoS ONE.

[61] Chung C.D., Liao J., Liu B., Rao X., Jay P., Berta P., Shuai K. (1997). Specific inhibition of Stat3 signal transduction by PIAS3. Science.

[62] Xiao H., Chung J., Kao H.Y., Yang Y.C. (2003). Tip60 is a co-repressor for STAT3. J. Biol. Chem..

[63] Muromoto R., Nakao K., Watanabe T., Sato N., Sekine Y., Sugiyama K., Oritani K., Shimoda K., Matsuda T. (2006). Physical and functional interactions between Daxx and STAT3. Oncogene.

[64] Tsuruma R., Ohbayashi N., Kamitani S., Ikeda O., Sato N., Muromoto R., Sekine Y., Oritani K., Matsuda T. (2008). Physical and functional interactions between STAT3 and KAP1. Oncogene.

[65] Tanaka T., Yamamoto Y., Muromoto R., Ikeda O., Sekine Y., Grusby M.J., Kaisho T., Matsuda T. (2011). PDLIM2 inhibits T helper 17 cell development and granulomatous inflammation through degradation of STAT3. Sci. Signal..

[66] Wu Z., Zhang X., Yang J., Wu G., Zhang Y., Yuan Y., Jin C., Chang Z., Wang J., Yang X. (2009). Nuclear protein IkappaB-zeta inhibits the activity of STAT3. Biochem. Biophys. Res. Commun..

[67] Hu B., Zhang K., Li S., Li H., Yan Z., Huang L., Wu J., Han X., Jiang W., Mulatibieke T. (2016). HIC1 attenuates invasion and metastasis by inhibiting the IL-6/STAT3 signalling pathway in human pancreatic cancer. Cancer Lett..

[68] Lin Y.M., Wang C.M., Jeng J.C., Leprince D., Shih H.M. (2013). HIC1 interacts with and modulates the activity of STAT3. Cell Cycle.

[69] Yao S., Xu F., Chen Y., Ge Y., Zhang F., Huang H., Li L., Lin D., Luo X., Xu J. (2017). Fbw7 regulates apoptosis in activated B-cell like diffuse large B-cell lymphoma by targeting Stat3 for ubiquitylation and degradation. J. Exp. Clin. Cancer Res. CR.

[70] Ren F., Su F., Ning H., Wang Y., Geng Y., Feng Y., Wang Y., Zhang Y., Jin Z., Li Y. (2013). SIPAR negatively regulates STAT3 signaling and inhibits progression of melanoma. Cell. Signal..

[71] Icardi L., Mori R., Gesellchen V., Eyckerman S., De Cauwer L., Verhelst J., Vercauteren K., Saelens X., Meuleman P., Leroux-Roels G. (2012). The Sin3a repressor complex is a master regulator of STAT transcriptional activity. Proc. Natl. Acad. Sci. USA.

[72] Zhang J., Yang J., Roy S.K., Tininini S., Hu J., Bromberg J.F., Poli V., Stark G.R., Kalvakolanu D.V. (2003). The cell death regulator GRIM-19 is an inhibitor of signal transducer and activator of transcription 3. Proc. Natl. Acad. Sci. USA.

[73] Huang Y., Yang M., Hu H., Zhao X., Bao L., Huang D., Song L., Li Y. (2016). Mitochondrial GRIM-19 as a potential therapeutic target for STAT3-dependent carcinogenesis of gastric cancer. Oncotarget.

[74] Tavecchio M., Lisanti S., Lam A., Ghosh J.C., Martin N.M., O’Connell M., Weeraratna A.T., Kossenkov A.V., Showe L.C., Altieri D.C. (2013). Cyclophilin D extramitochondrial signaling controls cell cycle progression and chemokine-directed cell motility. J. Biol. Chem..

[75] Richter K., Haslbeck M., Buchner J. (2010). The heat shock response: Life on the verge of death. Mol. Cell.

[76] Chatterjee S., Burns T.F. (2017). Targeting Heat Shock Proteins in Cancer: A Promising Therapeutic Approach. Int. J. Mol. Sci..

[77] Odunuga O.O., Longshaw V.M., Blatch G.L. (2004). Hop: More than an Hsp70/Hsp90 adaptor protein. BioEssays News Rev. Mol. Cell. Dev. Biol..

[78] Sun W., Xing B., Sun Y., Du X., Lu M., Hao C., Lu Z., Mi W., Wu S., Wei H. (2007). Proteome analysis of hepatocellular carcinoma by two-dimensional difference gel electrophoresis: Novel protein markers in hepatocellular carcinoma tissues. Mol. Cell. Proteom. MCP.

[79] Walsh N., O’Donovan N., Kennedy S., Henry M., Meleady P., Clynes M., Dowling P. (2009). Identification of pancreatic cancer invasion-related proteins by proteomic analysis. Proteom. Sci..

[80] Kubota H., Yamamoto S., Itoh E., Abe Y., Nakamura A., Izumi Y., Okada H., Iida M., Nanjo H., Itoh H. (2010). Increased expression of co-chaperone HOP with HSP90 and HSC70 and complex formation in human colonic carcinoma. Cell Stress Chaperones.

[81] Chao A., Lai C.H., Tsai C.L., Hsueh S., Hsueh C., Lin C.Y., Chou H.H., Lin Y.J., Chen H.W., Chang T.C. (2013). Tumor stress-induced phosphoprotein1 (STIP1) as a prognostic biomarker in ovarian cancer. PLoS ONE.

[82] Walsh N., Larkin A., Swan N., Conlon K., Dowling P., McDermott R., Clynes M. (2011). RNAi knockdown of Hop (Hsp70/Hsp90 organising protein) decreases invasion via MMP-2 down regulation. Cancer Lett..

[83] Minoguchi M., Minoguchi S., Aki D., Joo A., Yamamoto T., Yumioka T., Matsuda T., Yoshimura A. (2003). STAP-2/BKS, an adaptor/docking protein, modulates STAT3 activation in acute-phase response through its YXXQ motif. J. Biol. Chem..

[84] Ikeda O., Sekine Y., Mizushima A., Nakasuji M., Miyasaka Y., Yamamoto C., Muromoto R., Nanbo A., Oritani K., Yoshimura A. (2010). Interactions of STAP-2 with Brk and STAT3 participate in cell growth of human breast cancer cells. J. Biol. Chem..

[85] Porta R., Borea R., Coelho A., Khan S., Araujo A., Reclusa P., Franchina T., Van Der Steen N., Van Dam P., Ferri J. (2017). FGFR a promising druggable target in cancer: Molecular biology and new drugs. Crit. Rev. Oncol./Hematol..

[86] Fu H., Subramanian R.R., Masters S.C. (2000). 14-3-3 proteins: Structure, function, and regulation. Annu. Rev. Pharmacol. Toxicol..

[87] Morrison D.K. (2009). The 14-3-3 proteins: Integrators of diverse signaling cues that impact cell fate and cancer development. Trends Cell Biol..

[88] Simpson A.J., Caballero O.L., Jungbluth A., Chen Y.T., Old L.J. (2005). Cancer/testis antigens, gametogenesis and cancer. Nat. Rev. Cancer.

[89] Yang F., Zhou X., Miao X., Zhang T., Hang X., Tie R., Liu N., Tian F., Wang F., Yuan J. (2014). MAGEC2, an epithelial-mesenchymal transition inducer, is associated with breast cancer metastasis. Breast Cancer Res. Treat..

[90] Curioni-Fontecedro A., Nuber N., Mihic-Probst D., Seifert B., Soldini D., Dummer R., Knuth A., van den Broek M., Moch H. (2011). Expression of MAGE-C1/CT7 and MAGE-C2/CT10 predicts lymph node metastasis in melanoma patients. PLoS ONE.

[91] Koganti S., Hui-Yuen J., McAllister S., Gardner B., Grasser F., Palendira U., Tangye S.G., Freeman A.F., Bhaduri-McIntosh S. (2014). STAT3 interrupts ATR-Chk1 signaling to allow oncovirus-mediated cell proliferation. Proc. Natl. Acad. Sci. USA.

[92] Sun F., Xiao Y., Qu Z. (2015). Oncovirus Kaposi sarcoma herpesvirus (KSHV) represses tumor suppressor PDLIM2 to persistently activate nuclear factor kappaB (NF-kappaB) and STAT3 transcription factors for tumorigenesis and tumor maintenance. J. Biol. Chem..

[93] Zhou C., Cunningham L., Marcus A.I., Li Y., Kahn R.A. (2006). Arl2 and Arl3 regulate different microtubule-dependent processes. Mol. Biol. Cell.

[94] Chakravarty D., Tekmal R.R., Vadlamudi R.K. (2010). PELP1: A novel therapeutic target for hormonal cancers. IUBMB Life.

[95] Plowman G.D., Green J.M., Neubauer M.G., Buckley S.D., McDonald V.L., Todaro G.J., Shoyab M. (1992). The epithelin precursor encodes two proteins with opposing activities on epithelial cell growth. J. Biol. Chem..

[96] Zhou J., Gao G., Crabb J.W., Serrero G. (1993). Purification of an autocrine growth factor homologous with mouse epithelin precursor from a highly tumorigenic cell line. J. Biol. Chem..

[97] Lu R., Serrero G. (2000). Inhibition of PC cell-derived growth factor (PCDGF, epithelin/granulin precursor) expression by antisense PCDGF cDNA transfection inhibits tumorigenicity of the human breast carcinoma cell line MDA-MB-468. Proc. Natl. Acad. Sci. USA.

[98] Jones M.B., Michener C.M., Blanchette J.O., Kuznetsov V.A., Raffeld M., Serrero G., Emmert-Buck M.R., Petricoin E.F., Krizman D.B., Liotta L.A. (2003). The granulin-epithelin precursor/PC-cell-derived growth factor is a growth factor for epithelial ovarian cancer. Clin. Cancer Res. Off. J. Am. Assoc. Cancer Res..

[99] He Z., Ong C.H., Halper J., Bateman A. (2003). Progranulin is a mediator of the wound response. Nat. Med..

[100] Chung H.K., Yi Y.W., Jung N.C., Kim D., Suh J.M., Kim H., Park K.C., Song J.H., Kim D.W., Hwang E.S. (2003). CR6-interacting factor 1 interacts with Gadd45 family proteins and modulates the cell cycle. J. Biol. Chem..

[101] Kim S.J., Kwon M.C., Ryu M.J., Chung H.K., Tadi S., Kim Y.K., Kim J.M., Lee S.H., Park J.H., Kweon G.R. (2012). CRIF1 is essential for the synthesis and insertion of oxidative phosphorylation polypeptides in the mammalian mitochondrial membrane. Cell Metab..

[102] Bromberg J.F., Wrzeszczynska M.H., Devgan G., Zhao Y., Pestell R.G., Albanese C., Darnell J.E. (1999). Stat3 as an oncogene. Cell.

[103] Tomas A., Futter C.E., Eden E.R. (2014). EGF receptor trafficking: Consequences for signaling and cancer. Trends Cell Biol..

[104] Lo H.W., Hsu S.C., Hung M.C. (2006). EGFR signaling pathway in breast cancers: From traditional signal transduction to direct nuclear translocalization. Breast Cancer Res. Treat..

[105] Lo H.W., Hsu S.C., Ali-Seyed M., Gunduz M., Xia W., Wei Y., Bartholomeusz G., Shih J.Y., Hung M.C. (2005). Nuclear interaction of EGFR and STAT3 in the activation of the iNOS/NO pathway. Cancer Cell.

[106] Eckschlager T., Plch J., Stiborova M., Hrabeta J. (2017). Histone Deacetylase Inhibitors as Anticancer Drugs. Int. J. Mol. Sci..

[107] Gough D.J., Corlett A., Schlessinger K., Wegrzyn J., Larner A.C., Levy D.E. (2009). Mitochondrial STAT3 supports Ras-dependent oncogenic transformation. Science.

[108] Garama D.J., White C.L., Balic J.J., Gough D.J. (2016). Mitochondrial STAT3: Powering up a potent factor. Cytokine.

[109] Zhang Q., Raje V., Yakovlev V.A., Yacoub A., Szczepanek K., Meier J., Derecka M., Chen Q., Hu Y., Sisler J. (2013). Mitochondrial localized Stat3 promotes breast cancer growth via phosphorylation of serine 727. J. Biol. Chem..

[110] Shuai K., Liu B. (2005). Regulation of gene-activation pathways by PIAS proteins in the immune system. Nat. Rev. Immunol..

[111] Ogata Y., Osaki T., Naka T., Iwahori K., Furukawa M., Nagatomo I., Kijima T., Kumagai T., Yoshida M., Tachibana I. (2006). Overexpression of PIAS3 suppresses cell growth and restores the drug sensitivity of human lung cancer cells in association with PI3-K/Akt inactivation. Neoplasia.

[112] Junicho A., Matsuda T., Yamamoto T., Kishi H., Korkmaz K., Saatcioglu F., Fuse H., Muraguchi A. (2000). Protein inhibitor of activated STAT3 regulates androgen receptor signaling in prostate carcinoma cells. Biochem. Biophys. Res. Commun..

[113] Ikura T., Ogryzko V.V., Grigoriev M., Groisman R., Wang J., Horikoshi M., Scully R., Qin J., Nakatani Y. (2000). Involvement of the TIP60 histone acetylase complex in DNA repair and apoptosis. Cell.

[114] Hlubek F., Lohberg C., Meiler J., Jung A., Kirchner T., Brabletz T. (2001). Tip60 is a cell-type-specific transcriptional regulator. J. Biochem..

[115] Murr R., Loizou J.I., Yang Y.G., Cuenin C., Li H., Wang Z.Q., Herceg Z. (2006). Histone acetylation by Trrap-Tip60 modulates loading of repair proteins and repair of DNA double-strand breaks. Nat. Cell Biol..

[116] Moschos S., Varanasi S., Kirkwood J.M. (2005). Interferons in the treatment of solid tumors. Cancer Treat. Res..

[117] Stagg J., Loi S., Divisekera U., Ngiow S.F., Duret H., Yagita H., Teng M.W., Smyth M.J. (2011). Anti-ErbB-2 mAb therapy requires type I and II interferons and synergizes with anti-PD-1 or anti-CD137 mAb therapy. Proc. Natl. Acad. Sci. USA.

[118] Sisirak V., Faget J., Gobert M., Goutagny N., Vey N., Treilleux I., Renaudineau S., Poyet G., Labidi-Galy S.I., Goddard-Leon S. (2012). Impaired IFN-alpha production by plasmacytoid dendritic cells favors regulatory T-cell expansion that may contribute to breast cancer progression. Cancer Res..

[119] Zitvogel L., Galluzzi L., Smyth M.J., Kroemer G. (2013). Mechanism of action of conventional and targeted anticancer therapies: Reinstating immunosurveillance. Immunity.

[120] Yang X., Zhang X., Fu M.L., Weichselbaum R.R., Gajewski T.F., Guo Y., Fu Y.X. (2014). Targeting the tumor microenvironment with interferon-beta bridges innate and adaptive immune responses. Cancer Cell.

[121] Li R., Pei H., Watson D.K., Papas T.S. (2000). EAP1/Daxx interacts with ETS1 and represses transcriptional activation of ETS1 target genes. Oncogene.

[122] Chang C.C., Lin D.Y., Fang H.I., Chen R.H., Shih H.M. (2005). Daxx mediates the small ubiquitin-like modifier-dependent transcriptional repression of Smad4. J. Biol. Chem..

[123] Park J., Lee J.H., La M., Jang M.J., Chae G.W., Kim S.B., Tak H., Jung Y., Byun B., Ahn J.K. (2007). Inhibition of NF-kappaB acetylation and its transcriptional activity by Daxx. J. Mol. Biol..

[124] Puto L.A., Reed J.C. (2008). Daxx represses RelB target promoters via DNA methyltransferase recruitment and DNA hypermethylation. Genes Dev..

[125] Wen Z., Zhong Z., Darnell J.E. (1995). Maximal activation of transcription by Stat1 and Stat3 requires both tyrosine and serine phosphorylation. Cell.

[126] Wei J., Yuan Y., Jin C., Chen H., Leng L., He F., Wang J. (2012). The ubiquitin ligase TRAF6 negatively regulates the JAK-STAT signaling pathway by binding to STAT3 and mediating its ubiquitination. PLoS ONE.

[127] Romo-Tena J., Rajme-Lopez S., Aparicio-Vera L., Alcocer-Varela J., Gomez-Martin D. (2018). Lys63-polyubiquitination by the E3 ligase casitas B-lineage lymphoma-b (Cbl-b) modulates peripheral regulatory T cell tolerance in patients with systemic lupus erythematosus. Clin. Exp. Immunol..

[128] Kitamura H., Kanehira K., Okita K., Morimatsu M., Saito M. (2000). MAIL, a novel nuclear I kappa B protein that potentiates LPS-induced IL-6 production. FEBS Lett..

[129] Yamazaki S., Muta T., Takeshige K. (2001). A novel IkappaB protein, IkappaB-zeta, induced by proinflammatory stimuli, negatively regulates nuclear factor-kappaB in the nuclei. J. Biol. Chem..

[130] Rood B.R., Leprince D. (2013). Deciphering HIC1 control pathways to reveal new avenues in cancer therapeutics. Expert Opin. Ther. Targets.

[131] Davis R.J., Welcker M., Clurman B.E. (2014). Tumor suppression by the Fbw7 ubiquitin ligase: Mechanisms and opportunities. Cancer Cell.

[132] Ren F., Geng Y., Minami T., Qiu Y., Feng Y., Liu C., Zhao J., Wang Y., Fan X., Wang Y. (2015). Nuclear termination of STAT3 signaling through SIPAR (STAT3-Interacting Protein As a Repressor)-dependent recruitment of T cell tyrosine phosphatase TC-PTP. FEBS Lett..

[133] Lewis M.J., Liu J., Libby E.F., Lee M., Crawford N.P., Hurst D.R. (2016). SIN3A and SIN3B differentially regulate breast cancer metastasis. Oncotarget.

[134] Bansal N., David G., Farias E., Waxman S. (2016). Emerging Roles of Epigenetic Regulator Sin3 in Cancer. Adv. Cancer Res..

[135] Tammineni P., Anugula C., Mohammed F., Anjaneyulu M., Larner A.C., Sepuri N.B. (2013). The import of the transcription factor STAT3 into mitochondria depends on GRIM-19, a component of the electron transport chain. J. Biol. Chem..

[136] Boengler K., Hilfiker-Kleiner D., Heusch G., Schulz R. (2010). Inhibition of permeability transition pore opening by mitochondrial STAT3 and its role in myocardial ischemia/reperfusion. Basic Res. Cardiol..

[137] Avalle L., Camporeale A., Camperi A., Poli V. (2017). STAT3 in cancer: A double edged sword. Cytokine.

[138] Karin M., Cao Y., Greten F.R., Li Z.W. (2002). NF-kappaB in cancer: From innocent bystander to major culprit. Nat. Rev. Cancer.

[139] Kesanakurti D., Chetty C., Rajasekhar Maddirela D., Gujrati M., Rao J.S. (2013). Essential role of cooperative NF-kappaB and Stat3 recruitment to ICAM-1 intronic consensus elements in the regulation of radiation-induced invasion and migration in glioma. Oncogene.

[140] Kamitani S., Togi S., Ikeda O., Nakasuji M., Sakauchi A., Sekine Y., Muromoto R., Oritani K., Matsuda T. (2011). Kruppel-associated box-associated protein 1 negatively regulates TNF-alpha-induced NF-kappaB transcriptional activity by influencing the interactions among STAT3, p300, and NF-kappaB/p65. J. Immunol..

[141] Togi S., Shiga K., Muromoto R., Kato M., Souma Y., Sekine Y., Kon S., Oritani K., Matsuda T. (2013). Y14 positively regulates TNF-alpha-induced NF-kappaB transcriptional activity via interacting RIP1 and TRADD beyond an exon junction complex protein. J. Immunol..

[142] Sekine Y., Yumioka T., Yamamoto T., Muromoto R., Imoto S., Sugiyma K., Oritani K., Shimoda K., Minoguchi M., Akira S. (2006). Modulation of TLR4 signaling by a novel adaptor protein signal-transducing adaptor protein-2 in macrophages. J. Immunol..

[143] Tanaka T., Grusby M.J., Kaisho T. (2007). PDLIM2-mediated termination of transcription factor NF-kappaB activation by intranuclear sequestration and degradation of the p65 subunit. Nat. Immunol..

